# *N*-Desmethylclozapine, Fluoxetine, and Salmeterol Inhibit Postentry Stages of the Dengue Virus Life Cycle

**DOI:** 10.1128/AAC.01367-16

**Published:** 2016-10-21

**Authors:** Guruprasad R. Medigeshi, Rinki Kumar, Ekta Dhamija, Tanvi Agrawal, Meenakshi Kar

**Affiliations:** a Vaccine and Infectious Disease Research Center, Translational Health Science and Technology Institute, Haryana, India; b Department of Biotechnology, Jamia Hamdard, Hamdard Nagar, New Delhi, India

## Abstract

Around 10,000 people die each year due to severe dengue disease, and two-thirds of the world population lives in a region where dengue disease is endemic. There has been remarkable progress in dengue virus vaccine development; however, there are no licensed antivirals for dengue disease, and none appear to be in clinical trials. We took the approach of repositioning approved drugs for anti-dengue virus activity by screening a library of pharmacologically active compounds. We identified *N*-desmethylclozapine, fluoxetine hydrochloride, and salmeterol xinafoate as dengue virus inhibitors based on reductions in the numbers of infected cells and viral titers. Dengue virus RNA levels were diminished in inhibitor-treated cells, and this effect was specific to dengue virus, as other flaviviruses, such as Japanese encephalitis virus and West Nile virus, or other RNA viruses, such as respiratory syncytial virus and rotavirus, were not affected by these inhibitors. All three inhibitors specifically inhibited dengue virus replication with 50% inhibitory concentrations (IC_50_s) in the high-nanomolar range. Estimation of negative-strand RNA intermediates and time-of-addition experiments indicated that inhibition was occurring at a postentry stage, most probably at the initiation of viral RNA replication. Finally, we show that inhibition is most likely due to the modulation of the endolysosomal pathway and induction of autophagy.

## INTRODUCTION

Dengue virus (DENV) is a mosquito-borne pathogen that imposes maximum burdens on the economy and public health in large parts of the Americas, Southeast Asia, and Africa. According to recent estimates, close to 60 million symptomatic cases occur every year, and the overall economic burden of dengue is nearly $9 billion (1). Vaccine development efforts have resulted in the introduction of the first-ever dengue virus vaccine for human use in many countries, and other promising vaccine candidates are in advanced stages of clinical trials (2–4). Nevertheless, there is a lack of significant progress in antiviral development for dengue virus. A number of compounds have been identified as dengue virus inhibitors using *in vitro* and *in silico* approaches, but these compounds have not advanced to clinical trials in humans. Chloroquine, celgosivir, and balapiravir are the only drugs that have reached the clinical-trials stage; however, no results have been reported for these studies (ClinicalTrials.gov registration numbers NCT00849602, NCT01619969, and NCT01096576, respectively). Repurposing of drugs is one way of fast-tracking antiviral discovery, as it builds on preexisting knowledge of the candidate drug. In cases where the drug has been proven to be safe for human use for other conditions, this process helps to bring the drug to human use faster than a new candidate drug, which, according to industry estimates, takes 12 to 15 years from discovery to therapeutic use (5, 6). Drug-repurposing efforts have yielded the first antiretroviral, zidovudine (originally evaluated as an anticancer drug), approved for the treatment of HIV/AIDS. Gemcitabine, a nucleoside analog used for cancer treatment, has been shown to inhibit many viruses (7, 8). Screening efforts with 290 FDA-approved compounds led to the identification of 27 compounds with activity against Middle East respiratory syndrome coronavirus (MERS-CoV) and severe acute respiratory syndrome coronavirus (SARS-CoV) (9). These and other studies encouraged us to use this approach to screen the library of 1,280 pharmacologically active compounds (LOPAC^1280^) for anti-dengue virus compounds. LOPAC^1280^ covers most major pharmacologically relevant target classes, and many of them are approved as drugs for human use, thus providing an excellent platform for repurposing as antivirals. In this study, we report the screening of LOPAC^1280^ for dengue virus inhibitors using an imaging-based screening approach with Huh-7 cells. We identified three inhibitors from the library that specifically blocked early stages of dengue virus RNA replication. Our data further underscore the value and economic benefits of drug-repositioning efforts that could be harnessed to augment the antiviral pipeline.

## MATERIALS AND METHODS

### Cells and virus.

Huh-7 cells; DENV-2, Japanese encephalitis virus (JEV), and West Nile virus (WNV) isolates; virus growth conditions; and procedures for determining titers were described previously (10). (See the supplemental material for further details.)

### Inhibitor treatments.

Dengue virus infection and inhibitor treatment experiments were performed with Huh-7 and A549 cells, and viral titers in the culture supernatants were determined by plaque assays on BHK-21 cells. IC_50_ (50% inhibitory concentration) experiments were performed by using 2-fold dilutions of the inhibitor starting from an 8 μM concentration. A total of 30,000 cells were plated in a 48-well plate, infected with DENV-2 at a multiplicity of infection (MOI) of 3, and incubated with 2% Dulbecco's modified Eagle's medium (DMEM) containing inhibitors. Supernatants were collected at 24 h postinfection (p.i.), and viral titers were estimated by plaque assays on BHK-21 cells. For Western blotting, cell lysates were prepared as described previously (10), under the inhibitor treatment conditions described below, and the membranes were probed with antibodies recognizing DENV nonstructural protein 3 (NS3) (kind gift from Raj Bhatnagar), tubulin monoclonal (Developmental Studies Hybridoma Bank), calnexin (Thermo Fisher), and 78-kDa glucose-regulated protein (GRP78) (BD Biosciences) antibodies. For LC3 detection, an antibody that has a high specificity for the LC3-II form was used (LC3b [D11]; Cell Signaling Technology). Signals were detected by chemiluminescence. (See the supplemental material for a description of the flow cytometry experiment.)

### RNA transfection.

Twenty milliliters of the infected culture supernatant was concentrated by using Amicon Ultra concentrators with a 100-kDa cutoff (Merck-Millipore). RNA was isolated from the concentrated supernatant by using TRIzol-LS (Thermo Fisher Scientific). The aqueous phase obtained after the chloroform step was mixed with RNA binding buffer and processed for RNA isolation by using an RNA Clean and Concentrate kit (Zymo Research). A total of 400 ng of RNA was transfected into HEK293 cells by using Lipofectamine 2000 (Thermo Fisher Scientific). At 4 h posttransfection, medium containing dimethyl sulfoxide (DMSO) or inhibitors was added. At 72 h postinfection, total RNA was isolated as described above. The amount of DENV negative-strand RNA was estimated as described below.

### Quantitative real-time PCR (qRT-PCR).

Total RNA was isolated from cells by using either TRIzol or a Quick-RNA MicroPrep kit (Zymo Research). DENV RNA levels were estimated as described previously, using TaqMan one-step real-time PCR (Applied Biosystems) (10, 11). The amount of DENV negative-strand RNA was measured by cDNA synthesis with a forward primer followed by real-time PCR using a PrimeScript RT reagent kit with a genomic DNA (gDNA) eraser (TaKaRa). A β-actin primer-probe mix (Applied Biosystems) was used for normalization. Fold changes were calculated by the ΔΔ*C_T_* method.

### Immunofluorescence.

Huh-7 cells grown on coverslips in a 24-well plate were infected and treated with 4 μM inhibitors as described above. At 24 h p.i., cells were processed for immunofluorescence to visualize double-stranded RNA (dsRNA) as described previously (12). For assessing the effect of inhibitors on endolysosomal markers, Huh-7 cells were treated with the inhibitors as described above for 4 h. Cells were fixed and stained with antibodies against early endosome antigen 1 (EEA1) (BD Biosciences), lysosome-associated membrane glycoprotein 1 (LAMP-1) (Developmental Studies Hybridoma Bank), and LC3b followed by secondary antibodies conjugated with Alexa 488. Images were acquired with a 60× or a 100× objective by using an Olympus FV1000 or IX 83 inverted fluorescence microscope. Images were processed by background correction using Olympus FluoView software, and *z* projection images are shown.

### Statistical analysis.

GraphPad Prism software was used for all graphical representations and statistical analyses. All experiments were performed with two or three replicate samples, and results in each figure are a compilation of data from experiments performed at least two to three times. *P* values were estimated by a Mann-Whitney test or one-way analysis of variance (ANOVA).

## RESULTS

### High-throughput screening to identify inhibitors of DENV infection.

**FIG 1:**
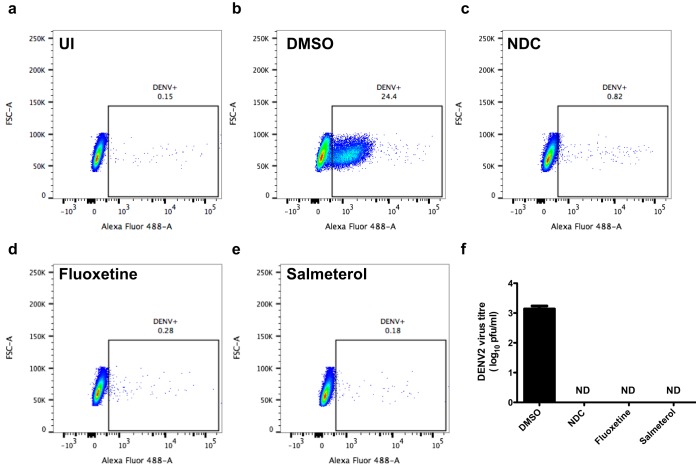
Inhibition of dengue virus infection is cell type independent. (a to e) A549 cells were mock infected (a) or infected with DENV at an MOI of 5 and treated with DMSO (b), 10 μM *N*-desmethylclozapine (NDC) (c), fluoxetine hydrochloride (d), and salmeterol xinafoate (e). DENV infection was analyzed by FACS analysis using DENV envelope-specific antibody. The DENV-positive cell population is gated. UI, uninfected; FSC, forward scatter. (f) Viral titers from the supernatants of the same cells were estimated by plaque assays. Error bars represent geometric means with 95% confidence intervals. ND, not determined.

We screened LOPAC^1280^ using high-throughput screening where both the reduction in the number of virus-infected cells as determined by a fluorescence imaging assay and the reduction in viral titers in the supernatant were used as endpoints. Huh-7 cells were infected with DENV-2 and treated with either 10 μM inhibitors or DMSO as a control. Cells were stained with DENV E antibody, and infected cells were visualized by using the Cell Analyzer imaging platform. We identified 30 compounds as primary hits that showed >50% inhibition of DENV infection. We confirmed the inhibitory activity of these compounds by a secondary screen using two independent assays measuring (i) viral titers in the supernatants by plaque assays and (ii) viral RNA levels at 24 h p.i. in cells treated with 10 μM inhibitors (see Fig. S1a and S1b in the supplemental material). Based on the secondary screen, we selected three inhibitors, *N*-desmethylclozapine, fluoxetine hydrochloride, and salmeterol xinafoate, which consistently showed >90% inhibition of both viral titers and viral RNA levels without cytotoxic effects (see Fig. S1c in the supplemental material). We next confirmed whether the inhibitors had any effect on flaviviruses closely related to DENV, such as Japanese encephalitis virus and West Nile virus. Huh-7 cells were infected with JEV and WNV, and virus titers were estimated at 24 h p.i. Surprisingly, none of the three inhibitors had any effect on either JEV or WNV titers (see Fig. S2a and S2b in the supplemental material). To further confirm that the observed inhibition of DENV infection is not specific to Huh-7 cells, we infected A549 cells with DENV-2 and treated the cells with inhibitors as described above. Viral titers from the supernatants were estimated by plaque assays at 24 h p.i. Cells were fixed and stained with DENV E antibody, and the percentage of DENV-positive cells was analyzed by fluorescence-activated cell sorter (FACS) analysis. We observed the complete absence of both virus-positive cells and viral titers in supernatants of cells treated with inhibitors compared to DMSO (Fig. 1a to f). These results suggest that the inhibitors acted on DENV in a cell type-independent manner. We next performed inhibition experiments with A549 cells infected with respiratory syncytial virus (RSV), a negative-strand RNA virus. The proportion of cells positive for RSV antigen was determined by FACS analysis with antibodies against the RSV fusion (F) protein at 24 h p.i. In contrast to DENV infection, none of the inhibitors had any effect on RSV infection, as observed by RSV positivity by FACS analysis in DMSO- and inhibitor-treated cells (see Fig. S3a to S3f in the supplemental material). Relative RSV genome levels were measured by RT-PCR, and similarly to the FACS results, there was no effect of inhibitors on RSV genome levels (see Fig. S3g in the supplemental material). To further verify the specificity of these inhibitors, we tested the effect of the three inhibitors on rotavirus (RV) infection. Caco-2 cells were infected with rotavirus, a double-stranded RNA virus, and treated with inhibitors as described above. Culture supernatants were collected at 18 h p.i., and viral titers were measured by plaque assays. As in the case of RSV, none of the inhibitors had any significant effect on rotavirus titers (see Fig. S4 in the supplemental material). These results indicate that the inhibitors are specific to DENV and may directly affect DENV infection by blocking one of the viral proteins or a target host pathway(s) that is specifically utilized by DENV. We next performed an inhibition experiment in C6/36 cells derived from Aedes albopictus mosquitoes. Surprisingly, the inhibitors had no effect on DENV titers in C6/36 cells (see Fig. S5 in the supplemental material), suggesting that the inhibitors may be targeting a factor(s) or pathway(s) that is expressed only in cells of human origin.

### Dengue virus inhibitors block viral replication.

**FIG 2:**
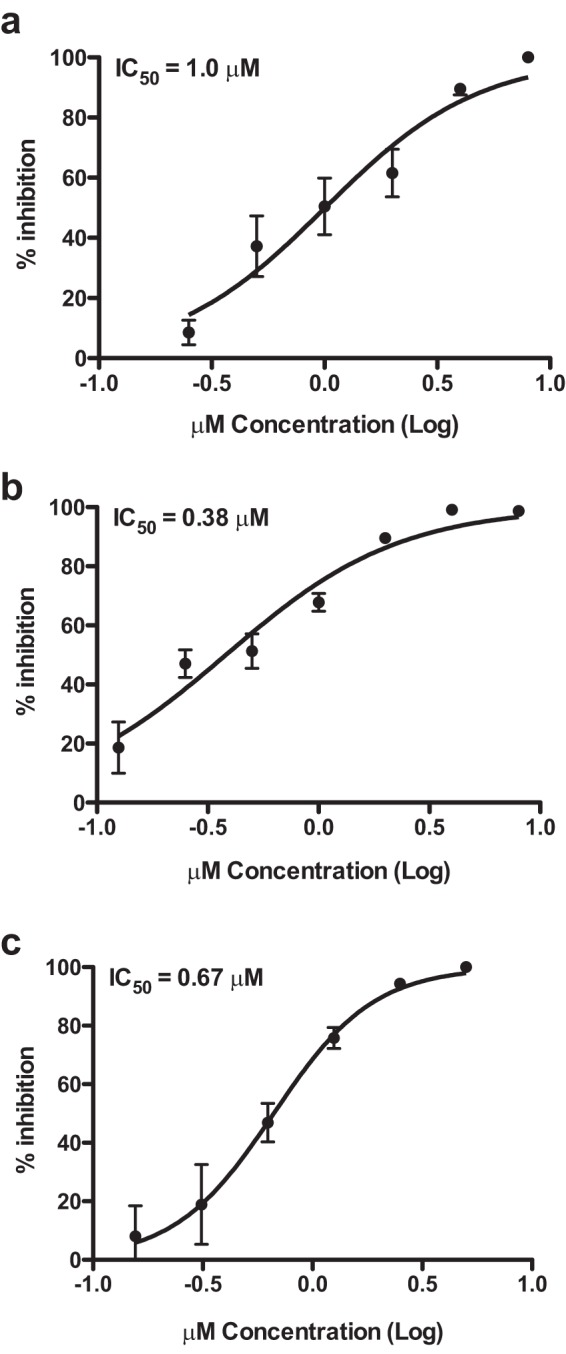
Determination of IC_50_s of dengue virus inhibitors. Huh-7 cells were infected with DENV-2 at an MOI of 3 and treated with 2-fold dilutions of inhibitors starting from a concentration of 8 μM. Viral titers in the supernatants were estimated by plaque assays at 24 h p.i. (a) *N*-Desmethylclozapine; (b) fluoxetine hydrochloride; (c) salmeterol xinafoate. Error bars represent means with standard errors of the means. IC_50_s of the respective drugs are indicated.

**FIG 3:**
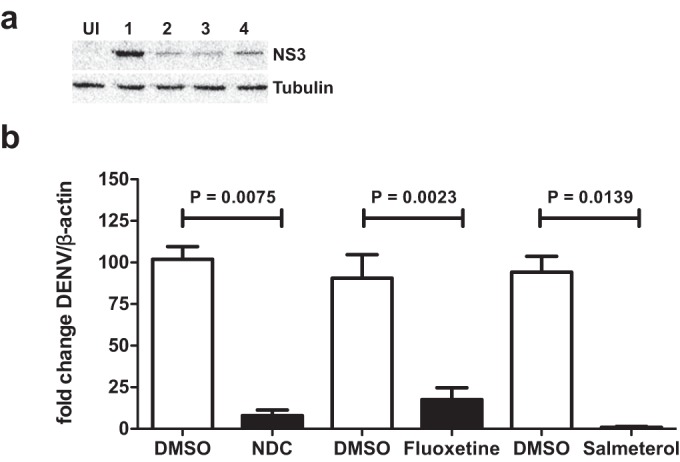
Dengue virus inhibition occurs at the level of RNA replication. (a) Huh-7 cells were mock infected (lane 1) or infected with DENV-2 at an MOI of 3 and treated with DMSO (lane 2) or 4 μM *N*-desmethylclozapine (lane 3), fluoxetine hydrochloride (lane 4), and salmeterol xinafoate (lane 5). Cell lysates were prepared at 24 h p.i., and DENV NS3 was detected by Western blot analysis. Equal loading of proteins is depicted by tubulin levels on the same blots. (b) Huh-7 cells were infected with DENV-2 at an MOI of 3 and treated with DMSO or 4 μM *N*-desmethylclozapine (NDC), fluoxetine hydrochloride, and salmeterol xinafoate. Total RNA was isolated, and the amounts of DENV RNA in the samples were estimated by qRT-PCR. Error bars represent means with standard deviations. *P* values were estimated by a Mann-Whitney test.

We next determined the IC_50_s of *N*-desmethylclozapine, fluoxetine, and salmeterol in Huh-7 cells infected with DENV-2. Cells were infected with DENV-2 and treated with increasing concentrations of each of the inhibitors, and viral titers were measured by plaque assays at 24 h p.i. The IC_50_s of *N*-desmethylclozapine, fluoxetine hydrochloride, and salmeterol xinafoate were 1 μM, 0.38 μM, and 0.67 μM, respectively (Fig. 2a to c). To further verify the stage of the viral life cycle that is affected by the addition of these inhibitors, we prepared total cell lysates at 24 h p.i. from cells infected with DENV and treated with DMSO or inhibitors. Viral infection was detected by performing Western blot analysis for DENV NS3. The levels of NS3 were reduced in cells treated with all three inhibitors compared to DMSO treatment, suggesting that the inhibitors act at a stage prior to viral protein translation (Fig. 3a). We next determined the DENV genome levels at 24 h p.i. by real-time PCR in total RNA isolated from DENV-infected cells treated with DMSO or inhibitors and found that DENV RNA levels were reduced by over 80% by all three inhibitors (Fig. 3b). As the inhibitors were added after virus entry into the cells in all the experiments, inhibition is likely to be occurring at the stage of either viral uncoating, translation of the viral RNA from the input virus, or RNA replication.

**FIG 4:**
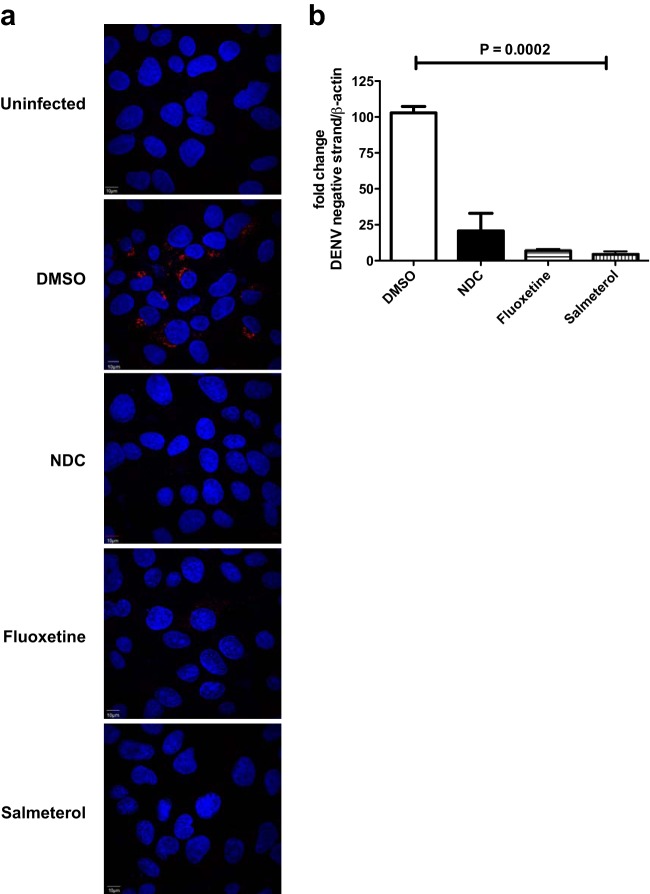
Inhibition of early stages of DENV RNA replication. (a) Huh-7 cells infected with DENV-2 and treated with DMSO or inhibitors (4 μM each) were fixed at 24 h p.i., and dsRNA intermediates were visualized by immunofluorescence using a primary antibody (J2) that recognizes dsRNA followed by a secondary antibody conjugated with Alexa Fluor 568. Nuclei were stained with 4′,6-diamidino-2-phenylindole (DAPI). (b) Huh-7 cells were infected with DENV-2 at an MOI of 3 and treated with DMSO or 4 μM inhibitors, and total RNA was isolated from cells at 24 h p.i. The amount of DENV negative-strand RNA was measured by qRT-PCR using primers that specifically amplify the negative strand during cDNA synthesis. Error bars represent means with standard deviations. The indicated *P* value was calculated by one-way ANOVA using a nonparametric Kruskal-Wallis test.

We next performed inhibition experiments in Huh-7 cells and staining for dsRNAs, which are intermediates in viral replication. dsRNA was detected only in infected cells treated with DMSO and not in any of the inhibitor-treated cells or mock-infected cells. (Fig. 4a). Furthermore, we measured the levels of negative-strand RNA intermediates at 24 h p.i. from DENV-infected cells that were treated with the inhibitors. *N*-Desmethylclozapine-treated cells showed a >75% reduction in negative-strand RNA levels, while both salmeterol- and fluoxetine-treated cells had <10% of the negative-strand RNA of DMSO-treated cells (Fig. 4b). These results demonstrate that all three inhibitors act at very early stages of dengue virus RNA replication.

**FIG 5:**
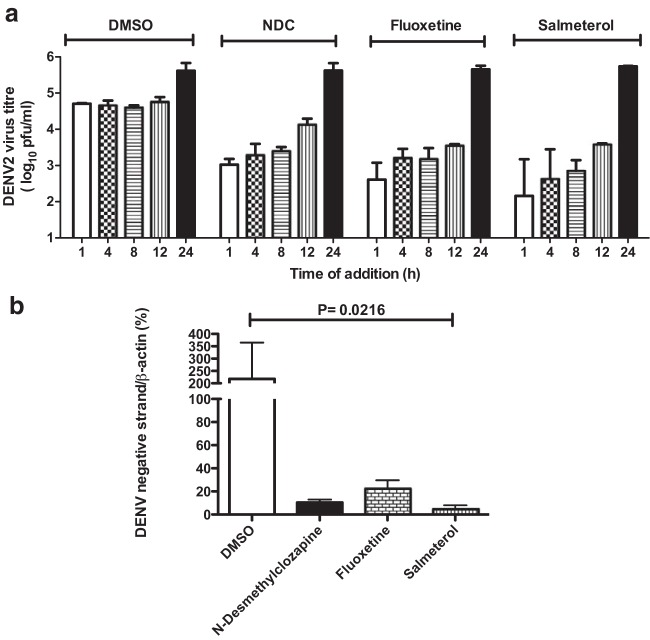
DENV inhibitors block early stages of the viral life cycle. (a) Huh-7 cells were infected with DENV-2 at an MOI of 3, and medium containing DMSO or 4 μM *N*-desmethylclozapine (NDC), fluoxetine, and salmeterol was added at the indicated time points postinfection. Viral titers in the supernatants were measured by plaque assays at 24 h p.i. For samples where inhibitors were added at 24 h p.i., viral titers were measured at 48 h p.i. Error bars represent geometric means with 95% confidence intervals. (b) HEK293 cells were transfected with 400 ng of viral RNA prepared from infected culture supernatants of C6/36 cells. Media containing DMSO or 4 μM inhibitors were added at 4 h posttransfection, and cells were collected at 72 h posttransfection for RNA isolation. The amount of negative-strand RNA was measured by qRT-PCR. Error bars represent means with standard errors of the means. The *P* value was calculated by one-way ANOVA using a nonparametric Kruskal-Wallis test.

We next assessed whether the inhibitors are capable of blocking postreplication stages of the viral life cycle. Cells were infected with DENV; inhibitors were added at 1, 4, 8, 12, and 24 h postinfection; and viral titers were measured by plaque assays at 24 h postinfection (for times of addition of 1, 4, 8, and 12 h) and at 48 h postinfection (for a time of addition of 24 h). The addition of inhibitors was inhibitory up to 12 h postinfection, whereas at 24 h postinfection, it had no effect (Fig. 5a). At 24 h postinfection, we expect later stages of the viral life cycle, namely, viral genome packaging, assembly, and egress, to be active compared to viral RNA replication; therefore, inhibitors that block viral RNA replication would have no effect at these stages of viral infection. Together, these data clearly suggest that all three inhibitors block early stages of the viral life cycle and are not able to prevent virus infection once the virus enters postreplicative stages of infection. To further demonstrate the effect of inhibitors at postentry stages, we transfected HEK293 cells with DENV-2 RNA prepared from infected cell culture supernatants, followed by incubation with medium containing DMSO or inhibitors. Total RNA was prepared from transfected cells at 72 h posttransfection, and the amount of negative-strand RNA was measured by RT-PCR. We observed a >80% reduction in the amount of negative-strand RNA in inhibitor-treated samples compared to DMSO treatment (Fig. 5b). These data clearly show that the inhibitors act at one or more stages post-virus entry, namely, membrane fusion, uncoating, or initiation of RNA replication.

### DENV inhibitors induce lysosome enlargement and autophagy.

**FIG 6:**
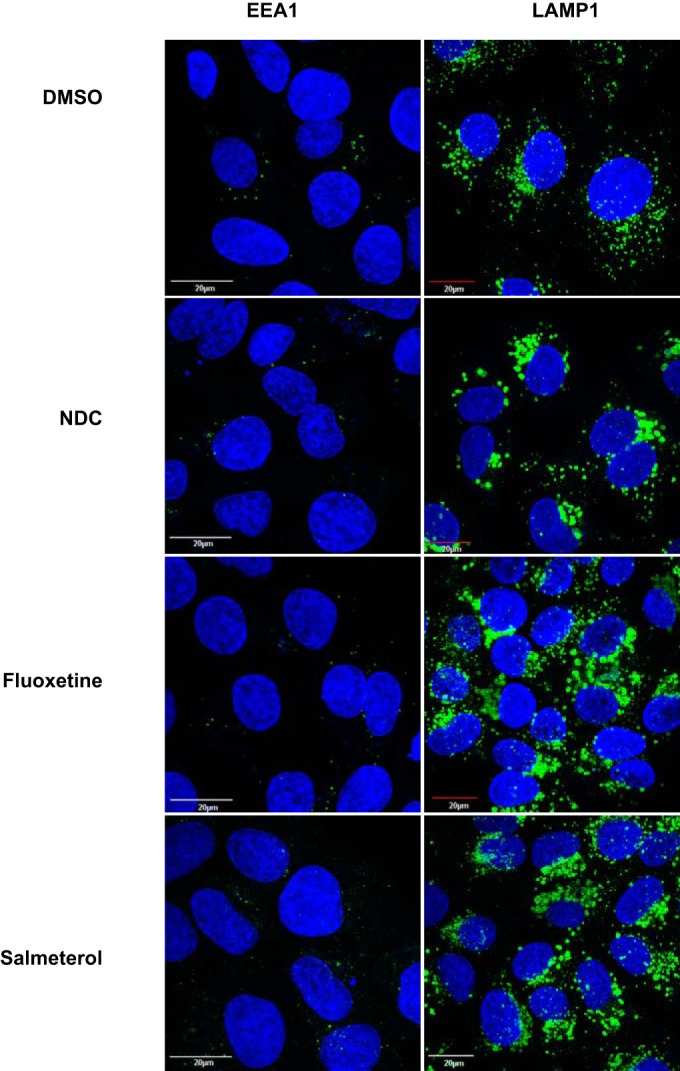
Effect of inhibitors on endolysosomal markers. Huh-7 cells were treated with DMSO or 4 μM *N*-desmethylclozapine (NDC), fluoxetine hydrochloride, and salmeterol xinafoate for 4 h; fixed; and stained with EEA1 and LAMP-1 primary antibodies followed by secondary antibodies conjugated with Alexa 488 (green). Nuclei are stained with DAPI (blue).

**FIG 7:**
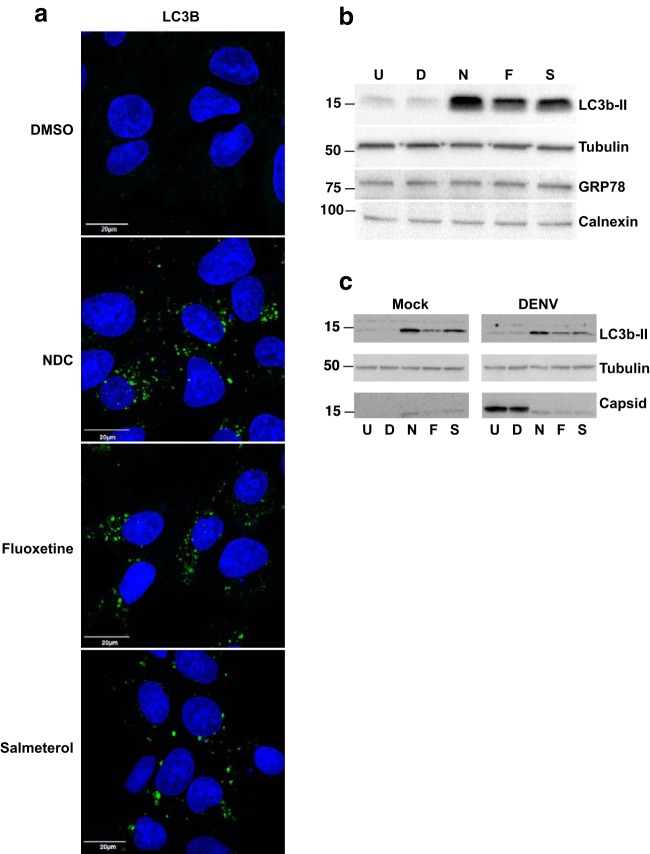
Dengue virus inhibitors induce autophagy. (a) Huh-7 cells were treated with DMSO or inhibitors for 4 h, fixed, and stained with an LC3b primary antibody followed by a secondary antibody conjugated with Alexa 488 (green). Nuclei were stained with DAPI (blue). (b) Huh-7 cells were untreated (U) or treated with DMSO (D) or 4 μM *N*-desmethylclozapine (N), fluoxetine (F), and salmeterol (S) for 4 h. Total cell lysates were analyzed by Western blotting for LC3b, tubulin, GRP75, and calnexin. The size of the prestained molecular weight marker (in thousands) is indicated. (c) Huh-7 cells were mock infected or infected with DENV-2 at an MOI of 3. Cells were further incubated with medium without inhibitors or treated with medium containing DMSO or 4 μM inhibitors for 24 h. Total cell lysates were analyzed by Western blotting for LC3b and tubulin. The LC3b blot was further probed with a DENV capsid antibody. The size of the prestained molecular weight marker (in thousands) is indicated on the left.

Internalized DENV has been shown to undergo membrane fusion in the late endosomal compartment (13). Therefore, we next investigated whether treatment with inhibitors had any effect on endolysosomal pathways. Huh-7 cells were treated with inhibitors for 4 h and stained with early endosome (EEA1) and late endosome/lysosome (LAMP-1) markers. Treatment with any of the three inhibitors had no effect on the EEA1 distribution; however, LAMP-1 staining was drastically different in cells treated with each of the inhibitors (Fig. 6). All three inhibitors induced a robust enlargement of late endosomes/lysosomes after 4 h of treatment, indicating the possible disruption of endolysosomal functions, thus affecting early stages of viral fusion, uncoating, and replication. In addition, we assessed whether treatment with inhibitors induced other stress response pathways such as the unfolded protein response or autophagy by immunofluorescence and Western blot analyses. We first verified the induction of autophagy by staining cells treated with inhibitors for LC3b, a well-established marker for autophagy induction. While LC3b staining was undetectable in DMSO-treated cells, treatment with the inhibitors clearly showed the presence of LC3b-positive structures (Fig. 7a). We next prepared total cell lysates from cells treated with inhibitors as described above to further verify the induction of autophagy. Cells treated with inhibitors showed a marked increase in the level of LC3b-II, a marker of autophagy, whereas the amount of cellular chaperones, namely, GRP78 or calnexin, remained unchanged (Fig. 7b). We next investigated whether the inhibitors were capable of inducing autophagy in the context of DENV infection. We observed that treatment with all three compounds but not DMSO led to the cleavage of LC3b, as detected by the LC3b-II band in both mock-infected and DENV-infected lysates (Fig. 7c). Note that DENV infection by itself did not lead to the induction of autophagy (as measured by LC3b-II detection) under these infection conditions. These data suggest that all three inhibitors of DENV replication induced autophagy, which may act as an antiviral response blocking early stages of dengue virus infection.

## DISCUSSION

We identified three compounds, *N*-desmethylclozapine, fluoxetine hydrochloride, and salmeterol xinafoate, as dengue virus replication inhibitors in a DENV screening assay. All three inhibitors reduced dengue virus infection with IC_50_s in the high-nanomolar/low-micromolar range. Cells treated with these inhibitors had low levels of dsRNA and negative-strand intermediates. This finding, along with the time-of-addition and RNA transfection experiments, demonstrated that the inhibitors interfered with early stages of dengue virus RNA replication. The effect was specific to dengue virus, as the inhibitors did not have any effect on other related flaviviruses, such as JEV and WNV, or on respiratory syncytial virus, a negative-strand RNA virus, and rotavirus, a virus with a double-stranded RNA genome. Finally, we showed that treatment with all three inhibitors led to lysosome enlargement and autophagosome formation, possibly affecting endolysosomal pathways that are specifically involved in early stages of the DENV life cycle. Although a number of studies have characterized direct-acting antivirals targeting dengue virus proteins (14–17), many cellular factors have also been identified and proposed as targets for antiviral development (10, 18–22). The two drugs identified in this study, *N*-desmethylclozapine and fluoxetine, regulate serotonin receptor signaling. However, a recent study demonstrated the inhibition of early stages of dengue virus replication by antagonists of dopamine receptor D4 but not serotonin receptor 2A (23). Therefore, the inhibitory effect of *N*-desmethylclozapine and fluoxetine is unlikely to be due to their direct effect on serotonin receptor signaling. A previous report showed inhibition of autophagosome turnover and impairment of lysosomal fusion by clozapine in cortical neurons from rat (24). Fluoxetine has been shown to induce the endoplasmic reticulum (ER) stress response and autophagy in certain cancer cell lines at 2- to 10-fold-higher concentrations than those used in our study (25, 26). Treatment of J774 murine macrophages for 3 days with various concentrations of fluoxetine showed no cytotoxicity but inhibited Mycobacterium tuberculosis growth by 50 to 75% in a concentration-dependent manner, primarily by inducing autophagy via the induction of tumor necrosis factor alpha (TNF-α) (27). The administration of fluoxetine also led to the induction of inflammatory cytokines in mouse frontal cortex (28). Similarly to those observations, we show that in Huh-7 cells, under our treatment conditions, there is induction of autophagy but not ER stress. We have not measured levels of inflammatory cytokines secreted after treatment with the inhibitors in our study. It is unlikely that inflammatory mediators would specifically alter DENV infection without affecting many other RNA viruses tested in our study. We speculate that the inhibitors identified in our study target pathways involved in endolysosomal biogenesis, thus affecting the fusion of internalized dengue virus virions with late endosomal membranes, or that the inhibitors target host factors that are required for dengue virus uncoating and/or the initiation of genome replication.

Previous studies have shown that both RSV and RV induced autophagy at later stages of infection (29, 30). RSV infection of dendritic cells induced autophagy, which was shown to be essential for the production of innate cytokines such as alpha interferon (IFN-α), interleukin-6 (IL-6), IL-12p40, and TNF-α (31). Abrogating the autophagic pathway negatively affects rotavirus titers (32), suggesting that while autophagy may restrict viral replication by the induction of innate immune responses in some cases, it may also be hijacked by some family of viruses, as it provides replication advantages. The role of autophagy in the life cycle of flaviviruses such as JEV and WNV has been controversial (33–38). In the case of DENV, a recent report demonstrated differential regulation of the autophagy pathway by DENV during the course of infection. Autophagy was shown to support DENV replication at early stages but to play an antiviral role at later stages, as DENV suppressed this pathway during later stages of infection (39). We did not observe an induction of autophagy under our infection conditions, most probably due to this suppressive effect. Single-particle-tracking studies have shown that DENV colocalizes with autophagosomes after endocytosis and undergoes fusion in late endosomes (40, 41). We speculate that treatment with *N*-desmethylclozapine, fluoxetine, and salmeterol leads to an alteration in autophagosome/lysosome biogenesis affecting early stages of the DENV life cycle. This observation also hints at differences in the pathways used by related flaviviruses at early stages of infection.

*N*-Desmethylclozapine is a major metabolite of clozapine and a potent 5-hydroxytryptamine 2 (5-HT2) serotonin receptor antagonist. Clozapine is a antipsychotic drug. Salmeterol is a β2-adrenergic receptor agonist and has anti-inflammatory properties (42). In conjunction with corticosteroids, salmeterol is currently prescribed as a bronchodilator for patients with chronic obstructive pulmonary disease (COPD) and persistent asthma. Fluoxetine is a selective serotonin reuptake inhibitor and is used as an antidepressant (43). There are very few reports on the antiviral activities of these compounds. Fluoxetine was shown previously to inhibit hepatitis C virus infection in Huh7.5 cells by blocking the production of reactive oxygen species and lipid accumulation (44). Fluoxetine was also identified as an inhibitor of coxsackievirus replication, and, as in our study, it was shown to inhibit viral replication but not viral entry or translation of the viral genome (45). Fluoxetine was identified in another antiviral screening study as an inhibitor of human enterovirus B and D replication (46). An evaluation of fluoxetine as a potential therapeutic option for enterovirus D68-associated acute flaccid myelitis was described previously (47). We have not tested the ability of *N*-desmethylclozapine and salmeterol to inhibit enteroviruses. It is likely that the inhibitors identified in our study may affect a pathway that is common to DENV and enteroviruses. Characterization of the host factors involved in early stages of DENV infection and its similarities with the enterovirus life cycle would help in identifying antiviral targets. Further work on *in silico* docking with known structures of dengue virus proteins and the generation of resistant viruses would provide clues with respect to the ability of these inhibitors to directly affect dengue virus replication. We believe that our study has provided new use for compounds that are approved for human use for other conditions. The safety profile and efficacy of these drugs need to be validated in pilot trials with dengue virus-infected patients. As these drugs would be used during the viremic phase of infection and for shorter periods in humans, we believe that the side effects observed with prolonged-dose regimens for other conditions may not be pertinent to viral infections. Finally, if these drugs are inhibiting dengue virus replication via cellular targets, the identification of molecular targets would help in the further development of safer and more potent antivirals against dengue virus.

## Supplementary Material

Supplemental material
