# Supplementary material for: N-Desmethylclozapine, Fluoxetine, and Salmeterol Inhibit Postentry Stages of the Dengue Virus Life Cycle
Source: Antimicrob Agents Chemother. 2016 Oct 21;60(11):6709–18. doi: 10.1128/AAC.01367-16 (PMC5075077; doi:10.1128/AAC.01367-16)
Supplement: Supplemental material [file supp_60_11_6709__index.html]

Supplemental material 

# *N*-Desmethylclozapine, Fluoxetine, and Salmeterol Inhibit Postentry Stages of the Dengue Virus Life Cycle

## Supplemental material

- Supplemental file 1 -

  Supplemental text and Fig. S1-S5

  PDF, 707K
