# Supplementary material for: N-Desmethylclozapine, Fluoxetine, and Salmeterol Inhibit Postentry Stages of the Dengue Virus Life Cycle
Source: Antimicrob Agents Chemother. 2016 Oct 21;60(11):6709–18. doi: 10.1128/AAC.01367-16 (PMC5075077; doi:10.1128/AAC.01367-16)
Supplement: Supplemental material [file AAC.01367-16_zac011165659so1.pdf]

SUPPLEMENTARY INFORMATION

METHODS

High throughput screening: Huh-7 cells were seeded into 96-well plate (2000 cells/well) and infected with 5 MOI of DENV-2. One hour post-infection (h pi) cells were washed twice with PBS and further cultured in DMEM with 2% FBS containing DMSO (vehicle control) or 10 µM inhibitors from LOPAC^{1280} (Sigma-Aldrich). 24 h pi cells were placed on ice and washed twice with ice-cold PBS and fixed with ice-cold methanol at -20° C for 30 min. Cells were washed twice with PBS followed by blocking and washing with PBS containing 0.2% BSA and stained with DENV-E primary antibody (4G2-Merck Millipore) for one hour followed by donkey anti-mouse Alexa-488 secondary antibody (Thermo Scientific) for 30 min. After secondary antibody incubation and washing, nuclei were stained by adding PBS containing DAPI for 10 min. Cells were washed and 100 µl PBS was added to each well. Plates were scanned with IN CELL ANALYZER 2000 (GE) and images were acquired for 6-9 fields (non-overlapping) per well. Analysis of acquired images was done with In Cell Analyzer Workstation 3.7.1 software (GE).

Cells and virus: A549 cells and Caco-2 were procured from European Collection of Authenticated Cell Cultures (ECACC) and cultured in Dulbecco's modified eagle medium with 10% fetal bovine serum, penicillin, streptomycin, glutamine

and non-essential amino acids. Respiratory syncytial virus (Long strain) was procured from American Type Culture Collection (ATCC-VR-26). Human rotavirus (Wa strain) and MA-104 cells were a kind gift from Dr. Gagandeep Kang. Rotavirus stock preparation and infection of Caco-2 cells (MOI of 0.5 pfu/cell) was performed as described previously except that serum free medium was used at all stages (1). For determination of viral titers, infected culture supernatants were activated by trypsin as described above, added on MA-104 cells were determined and continued incubation for one hour at 37° C. Virus inoculum was removed and overlay medium (MEM with 0.8% tracaganth and 0.5 µg/ml trypsin) was added. 3 days post-infection, overlay medium was removed and cells were fixed and stained as per the protocol followed for DENV.

For RSV stock preparation, HEp-2 cells (ATCC) were infected with an MOI of 0.1 pfu/cell for one hour in MEM containing 2% FBS. Cells were washed once with PBS, cultured in MEM-2% FBS and observed everyday for cytopathic effect and syncytial formation (3-4 days). Cells were scraped in 3-4 ml of culture medium and centrifuged at 1000 x *g* for 10 min at 4°C. Cell pellet was resuspended in 1 ml of MEM-2%FBS and passed through a 26G syringe attached to a one ml needle 10 times and centrifuged at 1000 x *g* for 10 min at 4°C. Multiple aliquots of the supernatant were prepared and stored at -80°C as stock virus. RSV titers were estimated by plaque assays on HEp-2 cells by following the same procedure as for DENV.

Flow Cytometry: For flow cytometry experiments, A549 cells were infected with DENV (5 MOI) or RSV (1 MOI), treated with 10 μM inhibitors. Cells were collected at 24 h pi by trypsinizaton. Cells were washed once to remove any traces of media and resuspended in 100 μl FACS buffer (0.25% FBS in 1X PBS) and stained with 1:1000 dilution of fixable viability stain-EF 780 (Becton Dickinson) for 15 minutes on ice. Cells were washed with FACS buffer and PBS once each and fixed in ice-cold methanol and incubated at -20° C for 20 minutes. Cells were washed with PBS twice and permeabilized using IMF buffer (2) overnight at 4° C. Cells were stained with mouse anti-RSV antibody (AbD Serotec) diluted in IMF buffer for 1 hour at 4° C followed by donkey-anti-mouse antibody conjugated with alexaflour-488 (Invitrogen) for 1 hour at 4° C. Appropriate unstained and secondary only controls were also processed in parallel. Cells were washed and acquired in BD FACS Canto-II and data was analysed using FlowJo software.

Cytotoxicity Assay: The cytotoxicity of the inhibitors was measured using CytoTox 96® Non-Radioactive Cytotoxicity Assay Kit (Promega G1780). Huh-7 cells were plated at a density of 30,000 cells/well in a 48-well plate. Next day, media containing 4-5 μM inhibitors were added and incubation continued for 24 hours. Untreated cells were lysed in lysis buffer provided in the kit and centrifuged to collect supernatant (positive control for 100% lysis). Amount of LDH release from all supernatants was measured as per the manufacturer's protocol.

REFERENCES

1. **O' Mahony J, O' Donoghue M, Morgan JG, Hill C.** 2000. Rotavirus survival and stability in foods as determined by an optimised plaque assay procedure. International Journal of Food Microbiology **61:**177-185.
2. **Haridas V, Rajgokul KS, Sadanandan S, Agrawal T, Sharvani V, Gopalakrishna MV, Bijesh MB, Kumawat KL, Basu A, Medigeshi GR.** 2013. Bispidine-amino acid conjugates act as a novel scaffold for the design of antivirals that block Japanese encephalitis virus replication. PLoS Negl Trop Dis **7:**e2005.

FIGURE LEGENDS

FIG S1. Primary validation of inhibitors of DENV infection from $LOPAC^{1280}$ screening: (a) Huh-7 cells were infected with 3 MOI of DENV-2 and treated with DMSO (vehicle control) or 10 μM inhibitors from the primary screen. Viral titers in the supernatant was estimated by plaque assay at 24 h pi and (b) total RNA was isolated from the cells to estimate DENV RNA levels by qRT-PCR. β-actin transcript levels were used for normalization. Dotted line indicates the 90% inhibition level. N-Desmethylclozapine, Fluoxetine hydrochloride and Salmeterol xinafoate treated samples are circled. (c) Cytotoxicity assays were performed in culture supernatants of cells treated with N-Desmethylclozapine, Fluoxetine

hydrochloride and Salmeterol xinafoate for 24 h. Error bars represent mean with SEM.

FIG S2. Effect of inhibitors on JEV and WNV infection. Huh-7 cells were infected with 3 MOI of JEV (a) or WNV (b) and treated with DMSO or 5 µM each of N-Desmethylclozapine, Fluoxetine hydrochloride and Salmeterol xinafoate respectively. Viral titers from the supernatants was estimated by plaque assays at 24 h pi. Error bars represent geometric mean with 95%CI.

FIG S3. Dengue inhibitors do not affect RSV infection: A549 cells were mock-infected (a) or infected with 1 MOI of RSV and treated with DMSO (b), 10 µM N-Desmethylclozapine (NDC) (c), Fluoxetine hydrochloride (d) and Salmeterol xinafoate (e) inhibitors. RSV infection was monitored by FACS using RSV-F antibody. RSV positive cell population is gated. (f) Virus positive cells (%) from two independent experiment is presented. (g) Total RNA was isolated from RSV infected, inhibitor-treated cells and the amount of RSV genome was estimated by qRT-PCR. Error bars represent mean with SD.

FIG S4. Dengue inhibitors do not affect rotavirus infection: Caco-2 cells were infected with 0.5 MOI of rotavirus and treated with DMSO or 20 µM inhibitors inhibitors. Viral titers from the supernatants was estimated by plaque assays at 18 h pi. Error bars represent geometric mean with 95%CI.

FIG S5. Effect of inhibitors on DENV2 infection in C6/36 cells. C6/36 cells were infected with 3 MOI DENV2 and treated with DMSO or 5 μM inhibitors. Viral titers in the supernatant was estimated by plaque assay at 24 h pi. Error bars represent geometric mean with 95%CI.

Figure S1

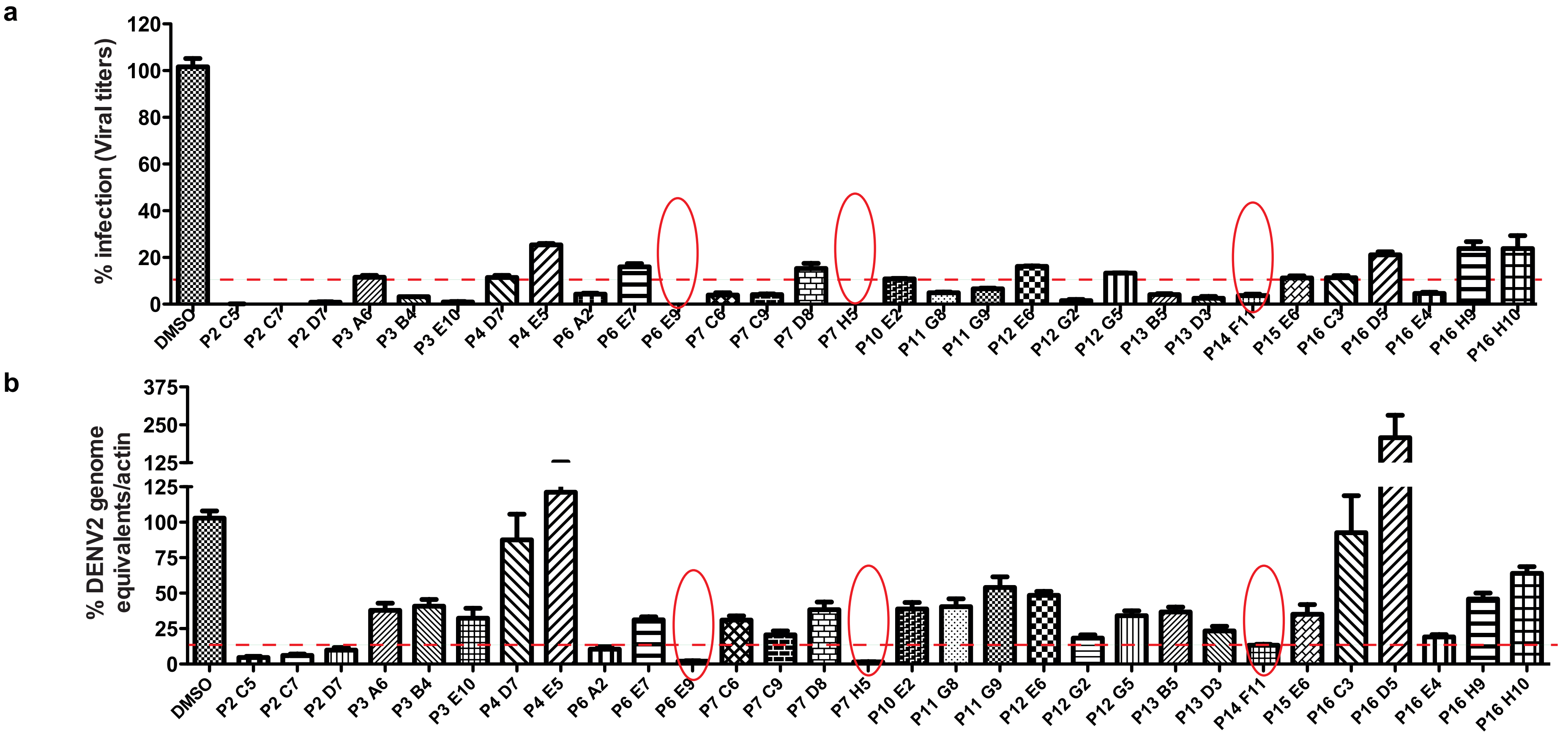

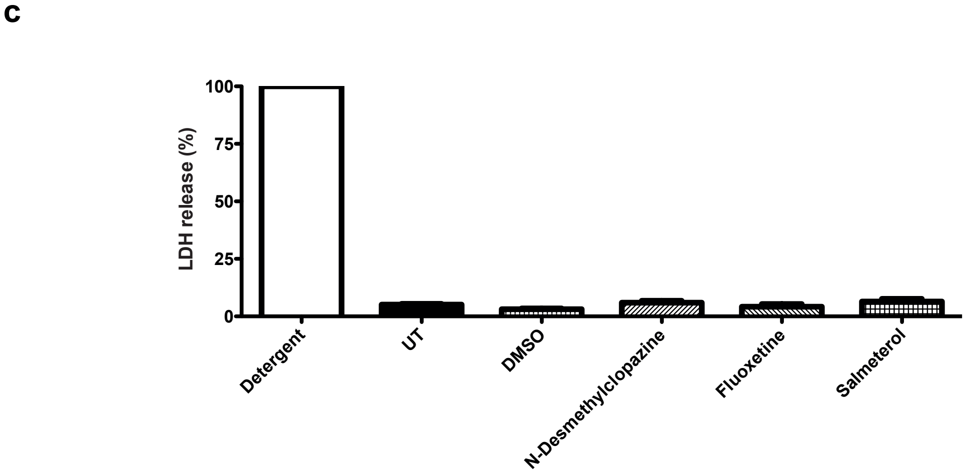

FIG S1. Primary validation of inhibitors of DENV infection from LOPAC1280 screening: (a) Huh-7 cells were infected with 3 MOI of DENV-2 and treated with DMSO (vehicle control) or 10 μM inhibitors from the primary screen. Viral titers in the supernatant was estimated by plaque assay at 24 h pi and (b) total RNA was isolated from the cells to estimate DENV RNA levels by qRT-PCR. β-actin transcript levels were used for normalization. Dotted line indicates the 90% inhibition level. N-Desmethylclozapine, Fluoxetine hydrochloride and Salmeterol xinafoate treated samples are circled. (c) Cytotoxicity assays were performed in culture supernatants of cells treated with N-Desmethylclozapine, Fluoxetine hydrochloride and Salmeterol xinafoate for 24 h. Error bars represent mean with SEM.

Figure S2

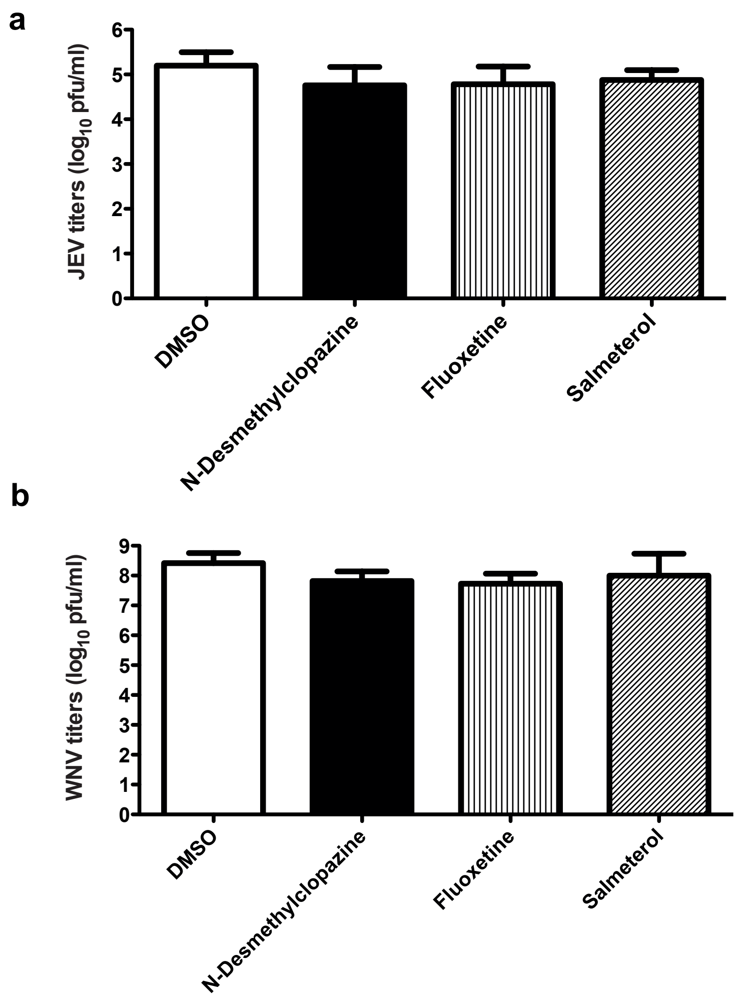

FIG S2. Effect of inhibitors on JEV and WNV infection. Huh-7 cells were infected with 3 MOI of JEV (a) or WNV (b) and treated with DMSO or 5 μM each of N-Desmethylclozapine, Fluoxetine hydrochloride and Salmeterol xinafoate respectively.

Figure S3

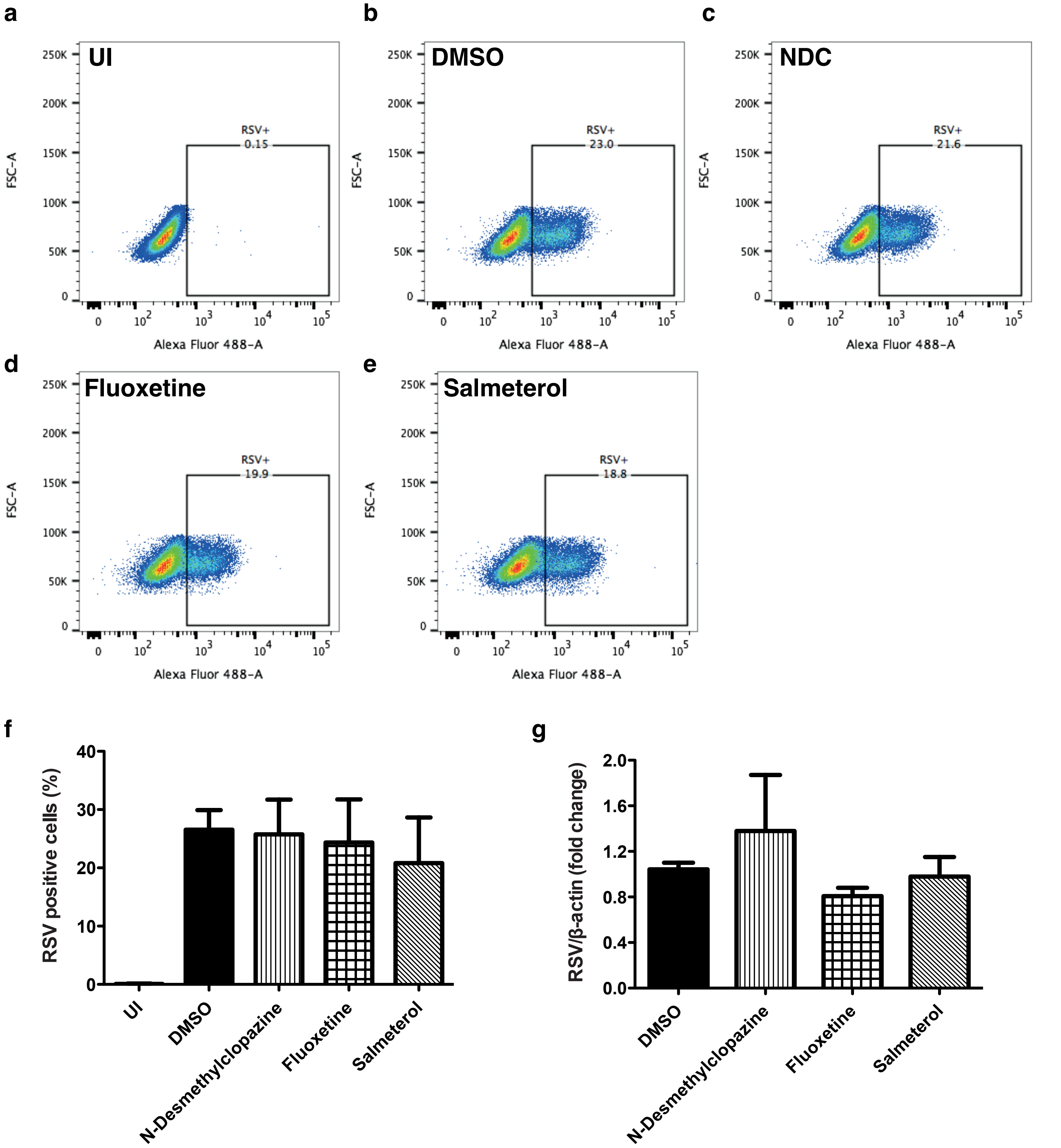

FIG S3. Dengue inhibitors do not affect RSV infection: A549 cells were mock-infected (a) or infected with 1 MOI of RSV and treated with DMSO (b), 10 μM N-Desmethylclozapine (NDC) (c), Fluoxetine hydrochloride (d) and Salmeterol xinafoate (e) inhibitors. RSV infection was monitored by FACS using RSV-F antibody. RSV-positive cell population is gated. (f) Virus positive cells (%) from two independent experiment is presented. (g) Total RNA was isolated from RSV-infected, inhibitor-treated cells and the amount of RSV genome was estimated by qRT-PCR. Error bars represent mean with SD.

Figure S4

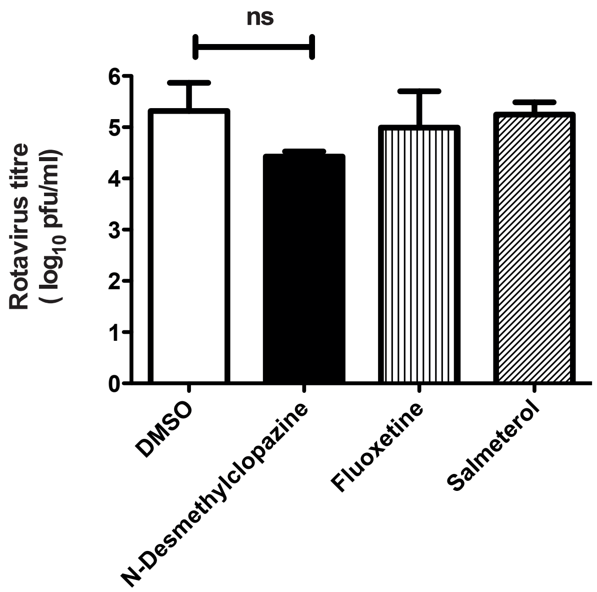

FIG S4. Caco-2 cells were infected with 0.5 MOI of rotavirus and treated with DMSO or 20 μM of inhibitors. Viral titers from the supernatants was estimated by plaque assays at 18 h pi. Error bars represent geometric mean with 95%CI.

Figure S5

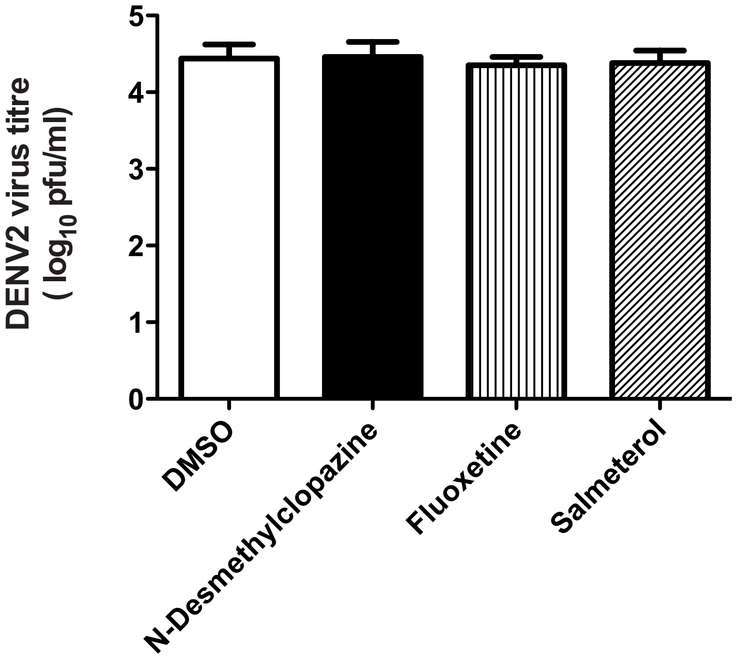

FIG S5. Effect of inhibitors on DENV2 infection in C6/36 cells. C6/36 cells were infected with 3 MOI DENV and treated with DMSO or 5 μM inhibitors. Viral titers in the supernatant was estimated by plaque assay at 24 h pi. Error bars represent geometric mean with 95%CI.
